# A Treatise on the Scrofulous Disease

**Published:** 1830

**Authors:** 


ART. VII.—*A Treatise on the Scrofulous Disease, by C. G. Hufeland, Physician to the King of Prussia, &c. Translated from the French of M. Bousquet, by Charles D. Meigs, M. D.—pp.* 215.—12*mo.*

The name of Hufeland, having become familiar to the physicians of the United States, through the medium of the periodical publications, this work, the first of his that has been republished in this country, we have no doubt will be very favourably received. A treatise had been published upon this subject by our author in 1797, when a premium having been offered by the Imperial Soc. of Naturalists, a new edition, corrected and improved by the observation and reading of twenty years was prepared, which took the prize.

As scrofula is principally confined to the two extremes of society, opportunities for acquiring an intimate acquaintance with it, fall to the lot of very few of our physicians. At the same time, the inveteracy of its different forms, the painful and incurable nature of many of them, the tenacity with which they adhere to the successive generations of a family, and the prospect of its increasing with the density of our population, and the growth of our manufacturing establishments, all conspire to render it a subject of peculiar interest.

Scrofula is a general disease of the lymphatic system, and consequently the local affections which pass by this name, are either symptoms of this general disease, or accidental determinations of it to particular organs. The complicated structure of the lymphatic system, its delicate though peculiar sensibility, and its intimate connexion with every part of the body, render it very liable to take on morbid action, from local injury, constitutional disease, or from sympathy with the diseases of other organs. So long as the general organic tone and sensibility of this system remain unimpaired, these local affections partake of the usual characters of sanguineous inflammation or irritation; and it is therefore under peculiar circumstances only, that they receive those modifications which entitle them to the appellation of scrofula.

*Of the nature or proximate cause of the Scrofulous Disease.*— The speculations of physicians upon the comparative influence of the solids and fluids in occasioning disease, have produced a very great difference of opinion upon this subject.— We do not think it consistent with sound philosophy to attach much importance to speculations of this kind. The presence of both solids and fluids is essentially necessary during every period of the animal growth and existence; and they exert so direct and immediate an influence upon the composition and healthful condition of each other, that neither can be diseased without producing a corresponding affection in the other.

The pathological changes, which take place in the minute ramifications of the vascular and nervous system, are too far beyond the observations of our senses to enable us to derive any advantage, from prosecuting our investigations in this direction, while the light, which physiology throws upon the reciprocal influence of the solids and fluids in those parts of the vascular system which lie more open to our observation, is just sufficient to enable the bold theorist to attribute the phenomena of disease to either. Both parties are led by experience to the same practical principles; both appeal to observation, and assume to themselves the exclusive merit of explaining the results of their common practice, upon rational principles. Speculations which are thus founded upon a common basis, must admit of some ground of compromise, and the unprejudiced enquirer will hesitate to admit principles, which tend to the separation of things so intimately associated, as are the solids and fluids of the animal æconomy, both in their healthy and pathological condition.

Entertaining these views, we cordially concur with our author, that in order to obtain a correct view of the pathology of scrofula, it is necessary to consider the structure, sensibility and functions of the lymphatic system as well as the nature and changing conditions of the fluid it conveys.

*General considerations on the lymphatic system.*—This system is composed of three orders of organs. 1. *Absorbent vessels properly so called*, an assemblage of a multitude of small, delicate, semitransparent, uneven vessels, provided with valves, arising by innumerable radicles from the membranes, and from the cellular tissue which is met with in every part of the body. 2. *The thoracic duct* in which the greater part of these vessels terminate. 3. *The lymphatic or conglobate glands*, small, oval, reddish bodies, composed of white vessels, blood vessels, and nerves, and distributed along the course of the lymphatics.

The principal functions of this system are two; 1. *Absorption.* This takes place, from the internal and external surfaces of the body, and from the intersticial spaces of the cellular membrane, and through these, from the substance of the organs of the whole body.

The object of this absorption is; (*a*) to repair the constant waste of the body; (*b*) the resorption of the lymph and other exhalations; (*c*) to absorb all heterogeneous particles, that they may be eliminated, and to remove the serous part of the secretions, when retained too long in their proper receptacles, as is especially the case, with the bile, the urine, and the semen, (*d*) to take up and transport morbific matter from one part of the body to another, as is often said to be the case with pus, (*e*) lastly, it is not improbable, that this set of vessels may exert a great influence in developing and modelling the organs, since they are peculiarly irritable at those periods when the growth of the body takes place most rapidly, and during the development of those organs whose functions are periodical, or exercised only during a certain period of our existence.

2. *Preparation and assimilation of the nutritive fluids.*

"The lymphatic system is not a simple passive highway, open to all those principles that are proceeding to enter into the great circulation. It is really in this system, that the organic molecules introduced into the body, begin to lose their heterogeneous qualities, and to approximate towards the character of animal matter; it is here they take the first step from the physical to the vital world; a species of preparation which disposes them to be clothed with the properties of the blood which they are destined to renew."

From this view it will appear that the matters that circulate will be:

1. Chyle produced from alimentary substances introduced into the primæ viæ.

2. Substances taken from the external surface of the body.

3. The substance of the organs themselves, which have become a prey to absorption.*

---

*We do not here wish to be considered as concurring with all the author's views. There are very strong reasons for believing that the functions of all the lymphatic vessels, whether arising from the alimentary canal or from the rest of the body are identical, and consist in absorbing and assimilating those substances which are intended for the renovation of the blood, while the absorption of extraneous substances which are again to be eliminated is left to the veins.

The exercise of these varied functions requires these vessels to possess a peculiar and very delicate sensibility, which is indeed shown to prevail by the number of nerves which enter into their composition, and by their proneness to take on inflammation, from irritation of the organs in which they have their origin.

This very imperfect abstract of our author's views, will serve to show how important the absorbent system is to the general health of the body, and how liable it is to be effected by disease, and the derangements of the organs. It supplies the organs with nourishment, and the quantity and quality of this, will very materially influence their structure and proper organization. It purifies the body, by removing heterogeneous or superfluous articles, and hence, from its imperfect action will result, emaciation, hypertrophy, serous infiltrations and morbid structure.

*Remote causes of the scrofulous disease.*—1. Every thing which may reduce the tone of the solids, and particularly that of the lymphatic system. 2. Whatever may exalt the irritability of this system, or blunt its sensibility. 3. Every thing that occasions chyle or lymph of a bad quality.

Causes of this class, do not merely give rise to a scrofulous taint, but if they act sufficiently long, give birth to the disease with all its characteristic symptoms.

They are 1. *Hereditary tendency.* The forms under which this constitutional disease manifests itself are; (*a*) the ophthalmia of infants; (*b*) cutaneous eruptions, ulceration and puriform discharges from the ears, all of which are scrofulous symptoms at that time of life; (*c*) glandular enlargements; (*d*) spina bifida; (*e*) induration of the cellular tissue. This disease is supposed by M. Hufeland to be scrofulous, partly from its analogy to the scrofulous diathesis, and partly because it was not noticed until the strumous constitution has become so common, and especially prevails where that constitution is most common.

2. *Sex and age.*—Children and women are the most liable to scrofula. In the latter, weakness and nervous susceptibility are promoted by the habits of the sex, and the great irritation which attend the development of their generative system, and in the former by the numerous predisposing causes, which accompany this period of life, and the irritability of the lymphatic system arising from their growth, and the development of their organs.

3. *Weakness of the parents.*—This cause especially operates when the organs of generation have been debilitated by venereal excesses, or unnatural practices. Children of a mother, and especially of a father far advanced in life, also bring into the world a scrofulous disposition, which is developed at an early period.

4. *Syphilis of parents.*—Syphilitic parents frequently produce children that very soon after birth present decided symptoms of the scrofulous taint, and it has been remarked that since the appearance of syphilis, this disease has become much more common, and most strikingly so, in those countries where the former is most prevalent. Nor is this surprising, since the lymphatic system is particularly affected in syphilis.

5. *Unwholesome aliment.* In this class are:

(*a*) "*Artificial lactation.*—Nothing is more injurious to the health than artificial suckling. I have almost always found that children brought up in this way were more or less disposed to the scrofulous disease."

Consumptive patients, bear milk perfectly fresh, better than after it has been suffered to stand an hour or two. It is in the former case still under the influence of the laws of vitality, and in the latter, have the chemical changes which now begin to take place rendered it less digestible? Whatever may be the rationale, the fact deserves attention. In continuation the author remarks:

"We commonly forget that most of the animals that furnish us with milk are purely herbiforous, while women derive their nutriment from each of the three kingdoms of nature. This difference in the nature of aliments produces an effect on the secretions. There is in the milk of animals something of a vegetable nature, which does not exist to the same amount in the milk of women. And hence the tendency to acidity, from which the chyle itself seems not to be wholly exempt, a tendency which is very common in children who are brought up by the hand."

"By the motion of its lips, the child that sucks its mother's breast occasions a flow of saliva into the mouth: this liquid mixes with the milk, enters into combination with its principles, which in this way acquire an inchoate animalization, that makes them more easy of digestion. The mixture of saliva with food, is a condition essential to a good digestion, not only in children, but in adults also.

"It results from what I have said above, that the milk of animals, and in general all artificial nourishments, furnishes a bad kind of chyle, and may, hence, become a predisposing cause of the scrofulous disease. However, as there are cases in which lactation is out of the question, I advise that the infant should be put to the teat of the animal, or at least, that it should take the milk while it is still warm, in order to diminish, as much as possible, the inconveniences which we attribute to artificial nourishment."

(*b*) *Impropriety, excess, and too much variety in diet.*—The sensibility of the stomach in children is adapted to a single article of diet, and the arrangements of nature cannot be interrupted with impunity. From the influence of this class of causes results a bad digestion, bad chyle, and lastly the scrofulous diathesis.

6. *Unwholesome air.*—The influence of a *cold and damp, an inconstant and variable, and an air highly carbonated, and charged with animal vapours,* is illustrated by the frequent appearance of the disease in low damp situations, on sea shores, in the vallies of the Alps, and in the lanes and cellars of large cities. Does light exert any influence over animal, as it does over vegetable life?*

7. *Every thing that weakens the digestive powers.*—The inconsiderate and premature use of tea and stimulating liquors. The abuse of medicines that exert a direct action on the primæ viæ, such as emetics and purgatives; tonics, astringents and opiates.

8. *Precocious studies.*—The culture of the body in the order of nature precedes that of the mind, and the latter, in youth, should never be suffered to interfere with the perfect growth and health of the body. This subject requires the greater attention, because parents ignorant of the fact that prema-

*The opinion is generally entertained, yet the fact that many young animals, during the period of their most rapid growth, live in entire darkness, and that others, as the hyena, seldom see the light of day, will throw some doubt upon this subject.

ture intelligence is a characteristic trait of scrofula, are delighted with the early manifestations of genius, and devote their whole care to the cultivation of a mind that is already too active for the feeble body which contains it.

9. To these causes we may add want of exercise and cleanliness; the abuse of heat and cold, depressing affections of the mind, and the too early exercise of the sexual organs.

*Occasional or exciting causes.*

1. *Development of the body, growth.*—We have already alluded to the irritation of the lymphatic system, which attended the progress of these changes, and which is evidenced by a proneness to inflammation, and by swellings of the glands, called *growing kernels*, that disappear after the body has completed its growth. This will serve to show why these periods are so favorable to the progress of a variety of scrofulous diseases. In some rare instances this irritation effects a cure of these diseases.

2. *The seasons.*—The spring increases the tendency to inflammatory diseases of every kind, and frequently gives activity to latent scrofulous affections. The summer, especially if rainy, produces upon some persons the same effects as the spring.

3. *Mechanical injuries.*

4. *Diseases of irritation.*—The diseases of the lymphatic system of the digestive tube and of the skin, have the most decided influence of this kind. The same effect however, will follow when the crisis or regular termination of any disease does not take place. The crisis of diseases of the skin is very generally incomplete, and the secretions continue long deranged, and are restored with difficulty. The crisis of diseases of the digestive organs, is frequently interrupted by excesses or improper food, and in all diseases the same result may follow the excessive use of evacuants, or the improper administration of tonics or anodynes.

From this review of the remote and exciting causes, the author infers that

1. *The scrofulous taint has its primitive seat in the solids* because, 1. An hereditary affection must necessarily consist in a modification of the solids. 2. The causes are either debilitating or irritating, and both these act upon the solids.

"2. *The scrofulous disease consists in a great degree of weakness and atony of the lymphatic system connected with a specific irritation of the same system, and in a peculiar alteration of the lymph.*

"Irritation and weakness,—such is the double character of the scrofulous disease. Irritation without weakness does not give us an exact idea of it; otherwise, all irritation of the lymphatic system would produce the scrofulous diathesis. Thus, variola and the scrofulous taint ought to be one and the same. The truth is, however, that in the latter the lymphatic system is at the same time too highly excited and too much debilitated, whether the weakness may have preceded the irritation, or whether it may have followed it.

"There is weakness, because:

"1. The weakest parents are most liable to give birth to scrofulous children.

"2. The affection attacks precisely that age and sex wherein weakness predominates; that is to say, children and women.

"3. Most of the essential causes are of a debilitating nature.

"4. The whole aspect of scrofulous persons bespeaks weakness and relaxation of the constitution.

"5. Tonics only are capable of destroying the scrofulous disposition.

That there exists at the same time a state or irritation is proved by the following:

"1. Most of the occasional causes are irritating; such are mechanical causes, the natural development of the organs, cold, &c.

"2. The slightest irritation of the lymphatic system sometimes suffices to bring to light the scrofulous taint.

"3. Most of its symptoms announce a disease of irritation.

"4. Antiphlogistics cause most of the symptoms to disappear, though they are incapable of destroying its germ."

3. *The proximate causes of the scrofulous disease exist in the fluids, as well as in the solids.*—In support of this opinion it may be urged, 1. That the fluids influence the tone and degree of cohesion of the solids, since they serve as the vehicle of the materials of which the latter are composed. (2) That the fluids are the natural stimuli of the vascular system, and there is a natural relation between the sensibility of the vessels, and the impression which the fluids make upon them. If, therefore, they make an insufficient impression the circulation languishes, and congestion and obstructions follow. But if on the contrary, the fluids are too acrid, the action is increased, and vascular irritation is produced. The lymphatic system is the more exposed to injuries from this source, inasmuch as they circulate the fluids by their own power independent of the action of the heart; and in addition, the peculiar sensibility which is necessary to the performance of the function of assimilation, opens a new avenue to disease.

4. *The affection of the lymphatic system which constitutes the scrofulous taint, alters the quality of lymph.*

The lymph may be variously affected. 1. It may be too thick and viscid, and not sufficiently irritating to keep up the necessary action of the vessels. 2. It may be too thin and watery, and destitute of the proper degree of animalazation. 3. It may be too irritating, as when it is loaded with acrid or saline principles, or when it is vitiated by the reabsorption of the secretions. 4. Or it may exist in too great abundance.

The causes capable of altering the composition of the fluids are; 1. The quality of the aliment. 2. The state of the digestive powers. 3. The qualities of the atmosphere. 4. The state of the secretions and of nutrition. 5. Imperfect assimilation.

There may be in this disease no absolute acrimony of the fluids, but merely a change of relation between the sensibility of the solids, and the impression made upon them by the fluids. The sensibility of the solids may either be increased or diminished, and disease will result, or the quality of the lymph which peculiarly adapts it to act as a stimulus to the vessels, may be varied, without its acquiring any positively irritating power. That the fluids of the body, are susceptible of variation in composition, will scarcely be denied by those who are acquainted with the diversity of their principles, and the elaborations they undergo; and if any doubts remain, the bare inspection of the blood drawn from persons laboring under different diseases, will be sufficient to remove them.

Besides the direct sources of vitiated lymph, which we have already assigned, there are others arising indirectly from the action of the vessels. It may be vitiated from stagnation in the vessels, or it may be hurried along without undergoing the requisite changes in the parts through which it has passed. Lastly, the vessels may be so debilitated that the lymph will be imperfectly animalized, and those changes will take place in it which arise from the laws of inanimate matter.

These are the changes which take place in the fluids of the lymphatic system. When the disease extends so as to attack other tissues; 1. The secretions become very irritating, and frequently inflame the parts over which they pass. 2. There is a tendency to the disposition of coagulable lymph. From this cause arise the enlarged lymphatic glands, joints, &c. and even the gibbosity of the vertebral column, usually arises from accumulation of lymph about the ligaments, and the bodies of the vertebræ and ribs. 3. The secretions frequently manifest a strong tendency to acidity, manifested in the green colour of the stools, the acid odour of the exhalations from the lungs, and of the perspiration. 4. When arrived at its highest degree, the disease is frequently attended with a scorbutic state of the fluids, indicated by colliquative sweats, repeated hemorrhages, and ulcers of a vinous colour. Lastly, when arrived at this stage, the author thinks that some forms of scrofula may become contagious.

*Description of the scrofulous diathesis.*

1. *External appearance.*—The appearance of the body which the author calls the *scrofulous physiognomy* is characteristic:

"1. A short thick neck. 2. The jaws rather stronger and broader than common. 3. The head rather large in proportion to the other parts of the body, especially the back part of the head. 4. Light coloured hair. 5. The face slightly bloated; its skin delicate, transparent, white, somewhat rosy. 6. Most commonly the eyes are blue, the pupils very large. This appearance often indicates a scrofulous state of the mesentery. 7. The upper lip rather thick.—This is one among the symptoms which do not mislead us; but I ought to remark that it is sometimes periodical. 8. The nose itself is often a little swelled, red, and shining. 9. The whole body appears to be fat and well nourished; but on a closer examination the flesh is found to be flabby and soft; it does not possess the resistance and elasticity which indicate health and vigor. 10. The belly is somewhat larger than it ought to be, although it may not have become as hard as it will be in the future progress of the affection. Sometimes it becomes very large from the very slightest causes.

"2. *Irregular development of the organs.*—One of the effects of the scrofulous disease is to retard the development of certain parts, and to hasten that of others.

"1. The development of the teeth, bones, and muscles, learning to walk and to talk, are either difficult, backward, or succeed each other in an irregular manner.

"2. The intellectual faculties, and those of the generative organs are, on the contrary, most commonly, very precocious."

Various ailments generally regarded as unimportant, are connected with the scrofulous diathesis, such as, 1. Frequent inflammation and derangement of the mucous surfaces.

2. The different cutaneous affections, and lastly, the various symptoms of derangement of the digestive organs.

3. *The scrofulous fever.*—A slow fever of an indeterminate character, that seems to form the transit from the scrofulous diathesis to the scrofulous disease.

*Characteristic symptoms of the scrofulous disease.*

1. *The first and most common is swelling of the lymphatic glands.* With the general character of these our readers are sufficiently acquainted. They are most frequent in the neck, after that in the axillæ, then those of the groin, and sometimes of the whole body. A single gland is rarely affected; they are often confounded together, and form an enormous tumour, or they become united so as to resemble a sort of chain. The internal tumours appear most frequently in the mesenteric glands causing marasmus; they are next to this most frequent in the lungs producing scrofulous consumption; they also appear in the liver, spleen, trachea, and sometimes in the brain.

2. *Cutaneous eruptions of different kinds, particularly about the head.* These are critical in their character, and divert the disease from the interior to the exterior surface of the body.

3. *Inflammations in organs which are liberally supplied with glands.* The eye is more especially affected. The well known disease called scrofulous opthalmia, thickening of the cornea, fistula lachymalis, are of this character. The ulceration of the tarsus, called stye, is an unfailing indication, in the opinion of the author, of a scrofulous diathesis.

4. *Mucous discharges* from the eyes, ears, the pulmonary, digestive, and genital organs.

5. *Scrofulous ulcers and lymphatic swellings.*—These last are indolent tumours of an irregular form, arising from depositions of lymph in the cellular tissue. To these we may perhaps add goitre, which often arises from local causes, but frequently is endemic in mountainous regions where scrofula abounds.

"*Period of disorganization.*—The third stage embraces, in the first place, those cases in which the scrofulous disease passes from its primary seat in the lymphatic system into the other tissues, and clothes them with unaccustomed forms; in consequence of which it is not always of easy recognition: and secondly, those in which it attacks such organs as are essential to life, and those whose disorganization menaces the near approach of death.

"1. *Mesenteric-atrophy*, (*marasmus.*)

"2. *White swelling of the joints.*—These generally depend on the scrofulous diathesis, and are ordinarily complicated with chronic inflammation of the bones and caries, or they degenerate into caries.

"3. *Spontaneous luxation.*—An affection peculiar to the hip, and almost always of a scrofulous nature.

"4. *Scrofulous dropsies.*—Hydrocephalus, both acute and chronic, frequently depends on this cause; and this is the reason why scrofulous children are more subject to it than any others. We have several times seen it take place after the suppression of a discharge from the eyes or ears, and after the repercussion of tinea, either occasioned by inconsiderate applications, or occurring spontaneously; and we have also known it to disappear after the return of these symptoms.

"5. *Scrofulous or tubercular consumption.*

"6. *Changes in the bones.*—Swellings of the bones known as spina ventosa, and caries are often effects of the scrofulous disease in its highest degree of intensity.

"7. *Scrofulous cancer.*—It is not a rare thing to see a scrofulous engorgement pass into the state of scirrhus, and even of cancer.—This is principally the origin of that humid cancer which is chiefly found on the lips.

"8. *Abdominal consumption*, (*tabes abdominalis.*)—Abdominal consumption differs from marasmus, because it does not occupy the same situation and developes itself in adult age, while the mesenteric atrophy is a disease peculiar to childhood.

"9. It cannot be doubted that rachitism derives its origin from the scrofulous taint, since it appears under the influence of the same causes, in individuals affected with the same taint, and, to use such an expression, at its expense; for it is a matter of observation that glandular engorgements and softenings of the bones mutually assist each other; though it is a fact that the glands are rarely affected secondarily in cases where rachitis is fully developed.

"10. *Nervous affections depending on the scrofulous taint.*—How often in the subjects of this taint have we seen cutaneous eruptions, swelled glands, &c. disappear suddenly, to give place to spasms, paralysis, hypochondriasis, and a hundred other such affections, which disappear in their turn on the restoration of the former symptoms!

"11. *Cretinism* is, in my opinion, the highest grade of the strumous constitution. In this case the infection is general; it is not a single part that is affected, but the whole body is scrofulous.

"It is observed, that the greatest number of deaf and dumb are found in scrofulous families. The same remark has been made of countries where the scrofulous diathesis is endemical."

*Treatment of scrofula.* The cure of the disease rests upon the following indications:

"1. To withdraw the patient from under the influence of the causes of the scrofulous diathesis; for a cure is impossible while these causes continue to act.

"2. Although acidities and mucous saburra oppress the primæ viæ evidently as effects of the scrofulous taint, nevertheless, these matters react on the cause which produces them, and oppose the cure; whence a necessity for keeping the bowels free, and for neutralizing the acids as fast as they are formed.

"3. To elevate the tone of the constitution, and especially that of the lymphatic system. This is the fundamental basis of the treatment.

"4. To regulate and reanimate the functions of the lymphatic system by allaying the irritation of which it is the seat.—

"Under this head we must arrange the class of tonics; because, the irritation of the lymphatic system being dependent on a state of weakness, we often find in tonics the most certain of our curative means.

"5. Wholesome aliments that are rich in nutritive principles, baths, a pure dry air, light, and sun-shine.

"6. To correct the direct effects of the scrofulous taint, such as engorgements of the glands, acrimony of the lymph, &c.

"In this case we ought carefully to inquire, whether the scrofulous disease is accompanied with irritation or asthenia: for although the latter ordinarily predominates, it sometimes happens, nevertheless, that the lymphatic system is highly irritated either in its whole, or partially. We must under such circumstances recur to the use of antiphlogistics until the irritation shall have been subdued. Frequently in these very cases, as for example in violent opthalmias, it is necessary to make use of local bleedings.

"We have seen above, that the exterior symptoms of scrofula alternate, so to speak, with the internal, and vice versa; so that the one may be regarded as a crisis of the other. Hence the important precept, to favour, as much as possible, the tendency of the scrofulous blemish to the exterior of the body;—for this is the means to arrest its progress, to preserve the internal organs from its attack, to render the general treatment more successful. Among the means which answer this indication, the chief are baths, stimulants to the skin, and issues. I could cite a multitude of instances in support of this truth, but one will be sufficient."

*Diatetic treatment.* Our limits will not permit us to do more than give a very cursory view, which we regret the less, as there is no deficiency of information upon this point. It consists of; 1. food, light and digestible, of vegetables and lean meat; 2. a warm, dry, and open air, and if practicable in the country.

3. *Exercise.* This will be the more effectual, if voluntary and elicited in the way of amusement.

4. *Attention to cleanliness.* This is to be effected:

"1. By daily ablutions of the whole body with fresh water.

"2. By ordering one or two tepid baths every week: but we shall recur to this measure.

"3. By frequently changing the body linen.

"1. In addition to the fact that there is no better means of keeping the skin clean, baths have the further advantage of facilitating both absorption and exhalation.

"2. Another property which cannot be denied them is that of causing an equal distribution of the forces, by means of the gentle excitement they produce over the whole surface of the body, by enlivening the sensibility, re-establishing the equilibrium of the fluids, soothing the excessive irritation, facilitating the absorbing operations, and regulating the functions of the lymphatic system.

"3. Even when scrofula is in full vigour, baths are very useful in preventing metastases, and for diminishing the lymphatic acrimony.

"*Pharmaceutical Treatment.*—We shall begin by laying down rules, the knowledge of which seems to be essential to a successful treatment of scrofula.

"*First rule.* No disease demands more patience on the physician's part than this; being by nature essentially chronic, time is one of the necessary conditions for its successful treatment.

"*Second rule.* Time and the natural development of the organs are two precious elements which ought to be taken into the account in the treatment of scrofulous affections. It has already been shown how, by means of this development, and by the natural revolutions of the lymphatic system, this affection may either cure itself or become easily curable.

"*Third rule.* Spring is, without contradiction, the most favorable season for combating the scrofulous diathesis.

"*Fourth rule.* Never mistake the suppression of glandular tumours, and of other local affections for the cure of the scrofulous diathesis.

"*Fifth rule.* Do not believe in the existence of a specific remedy for the scrofulous taint; a disease which acknowledges many causes, and which is so identified with the *constitution* that *it* must be entirely renovated before it can be rescued from it: such an affection cannot be cured by a single remedy.

"*Seventh rule.* The curative means must be occasionally varied in order to obviate the inconveniences arising from habit. All medicinal substances act with renewed vigor, whenever they are exhibited under new forms; a standing rule which cannot be too often repeated.

"After having laid down the rules which should direct us in the administration of the pharmaceutic means, I shall now proceed to speak of these means:

"1. *Emetics.* Among the means employed in curing the scrofulous diathesis, emetics deserve a distinguished place. To the advantage of cleansing the first passages, a matter of importance in this disease, they add that of exciting absorption and strongly agitating the lymphatic system.

"Although emetics are never formally contra-indicated in scrofula, I nevertheless rarely employ them where there is manifest atony of the digestive organs. I prefer, in scrofulous and pituitous habits, a mixture of one or two grains of tartar emetic with twelve grains or a scruple of ipecacuanha, in half an ounce of oxymel of squills and an ounce and a half of water."

2. *Purgatives.* This class of remedies is particularly indicated in scrofula, by their direct operation in evacuating the alimentary canal of its irritating contents, and indirectly by their influence over the absorbent system. The author proscribes the neutral salts entirely. He relies principally upon jalap, and the resinous purgatives, which by their resolvent power, counteract the debility that might be expected to follow their operation. Aloes are indicated where there is general debility with languor, and engorgement of the abdominal organs, but should be carefully avoided in all cases of inflammatory or nervous irritation. Rhubarb is an excellent purgative and tonic which may be used more freely than aloes in inflammatory cases.

3. *Antimony* in small doses exert a peculiar influence over the lymphatic system. It restores all the secretions of the skin, the liver and the lungs, promotes absorption, and the resolution of glandular engorgements, and reduces inflammatory action. It is particularly useful when the skin does not perform its functions properly, or where there are cutaneous eruptions and ulcers. When too long continued, it has a tendency to debilitate the system, and especially the stomach and bowels. Where the remedy is not contra-indicated, its injurious effects may be obviated by varying the preparation, or by the combination of narcotics and tonics.

4. *Mercury*. The long continued use of mercury debilitates the system, and if glandular engorgements are in a state of high irritation, it hastens their suppuration. If irritation prevails therefore, it should be diminished by purgatives or local bleeding, or if there be much debility, its effects should be counteracted by tonics, aromatics and anodynes; and salivation, under all circumstances, should most carefully be avoided.

"1. Mercury may be given with advantage in all the forms of the scrofulous disease, but principally in cutaneous eruptions, engorgements, lymphatic infiltrations, chronic phlegmasias, and more particularly still in the opthalmias and nervous affections dependent on the scrofulous taint.

"2. But too much circumspection cannot be used in those individuals, who by the very structure of their bodies appear to be disposed to phthisis, in those who are threatened with scurvy, as well as in all cases of weakness of the alimentary canal, and in all individuals subject to abundant hemorrhages.

"The combination of sulphur and antimony with mercury deprives the latter of such of its properties as are too irritating, favours its determination to the surface, while salivation is less to be dreaded from it."

5. *Muriate of baryta*. This remedy acts by a peculiar irritation on the alimentary canal, the conglobate and mesenteric glands, and removes their obstructions; it increases the secretions. It is peculiarly efficacious in affections of the skin, ulcers and ophthalmias, and it does not like mercury and antimony, weaken the digestive organs.

"It is especially adapted to cases where the lymphatic system is irritated, an irritation which iron, mercury, bark, &c. would not fail to aggravate; also, when scrofula has seized upon organs of a very irritable nature, such as the eyes, lungs, &c.

"In the scrofulous affections of the lungs, which it is so easy to exasperate, and where even calomel is too irritating, I look upon the muriate of baryta as one of the best medicines within our reach."

6. *Muriate of lime.*

"This article enjoys nearly the same properties with the one we have just been considering, except that it is more irritating; indeed it more decidedly increases both the sweat and urine. It requires therefore more precaution in its exhibition."

7. *Bark and the astringent tonics.* The weakness accompanying this disease appears to indicate this class of remedies, where there is no inflammation, or where the irritation which produces the inflammation arises from weakness. Its administration is frequently rendered more safe and efficacious by the combination of antispasmodics, narcotics and antiphlogistics.

8. *Preparations of iron.*

"1. Iron, as I just now said, is more astringent than bark. It is not adapted to those hard and immoveable indurations in which the cohesion is already too great. 2. As an excitant of the sanguiferous system, it is clear, that it would aggravate the condition of the patient, provided that there were the slighest tendency to inflammation. 3. When the lungs are irritated, when they are the seat of some minute glandular swellings, if there be cough, or flying pains in the breast, be careful to avoid the martial preparations, for they would bring on the pulmonary consumption, with which such patients are already menaced. 4. The same remark applies to cases in which an internal suppuration is going on. 5. But, is the patient of a light complexion? Has he a lax fibre? Does his whole constitution announce weakness? Is he disposed to the serous diathesis? The preparations of iron work miracles in such cases."

9. *Narcotics.*

"Opium, hyosciamus, cicuta, belladonna, bitter sweet, and all the articles which compose the class of narcotics, are very much employed by modern physicians.

"Their immediate and essential effect is to diminish the sensibility, and even, in some degree, the irritability also. Now, the essence of the scrofulous vice consists, as we have before said, in a simultaneous state of irritation and weakness of the lymphatic tystem. Therefore, narcotics may suit very well as far as regards the first of these circumstances, but not so well as to the second; and as, in general, the irritation depends in this case on weakness, it follows that these medicines can only produce a temporary relief, even in the most fortunate circumstances. I repeat, that tonics alone are capable of effecting a solid and durable cure.

"But when the irritation is primitive, or at least when it is not a consequence of weakness, every thing capable of diminishing sensibility may be useful. In this view the narcotics deserve a distinguished place in the treatment of this sort of irritation."

